# Fatty acid compositions, free radical scavenging activities, and antioxidative enzyme activities of high-preference and low-preference beef cuts of Hanwoo (*Bos taurus coreanae*) cows

**DOI:** 10.5713/ajas.18.0069

**Published:** 2018-07-26

**Authors:** Sang-Ho Moon, Eun-Kyung Kim, Se Young Jang, Yujiao Tang, Hye-Jin Seong, Yeong Sik Yun, Sanguk Chung, Mirae Oh

**Affiliations:** 1 Department of Food Bio Science, College of Biomedical and Health science, Konkuk University, Chungju 27478, Korea; 2 Institute of Livestock Environmental Management, Daejeon 34068, Korea; 3 School of Bio-sciences and Food Engineering, Changchun University of Science and Technology, Changchun 130600, China; 4 Environmental Microbial and Food Safety Laboratory, Agricultural Research Service, United States Department of Agriculture, Beltsville, MD 20705, USA

**Keywords:** Beef, Polyunsaturated Fatty Acid, Free Radical Scavenging Activity, Antioxidative Enzyme Activity, Hanwoo

## Abstract

**Objective:**

This study compared fatty acid compositions and antioxidant activities of high-preference cuts (loin, tenderloin, and rib) and low-preference cuts (brisket, topside, and shank) of Hanwoo (*Bos taurus coreanae*) cows to obtain potentially useful information for promoting the consumption of various low-preference cuts.

**Methods:**

Individual 500 g samples of fresh beef were collected from each of the six cuts from 10 Hanwoo cows (quality grade 1) and immediately freeze-dried. The dried samples were evaluated for fatty acid composition, free radical scavenging activities (hydroxyl, alkyl, and 2, 2′-diphenyl-1-picrylhydrazyl [DPPH] radical), and antioxidative enzyme activities (glutathione peroxidase [GPx], glutathione-S-transferase [GST], and superoxide dismutase [SOD]).

**Results:**

The percentages of total polyunsaturated fatty acids were significantly higher in low-preference cuts than in high-preference cuts (p<0.05). Hydroxyl, alkyl, and DPPH radical scavenging activities were significantly higher in low-preference cuts than in high-preference cuts (p<0.05). In addition, the activities of antioxidant enzymes, such as GPx, GST, and SOD, were significantly higher in low-preference cuts compared with high-preference cuts (p<0.05).

**Conclusion:**

These results may influence consumers to include more low-preference cuts in their selections based on the nutritional facts, which could help to balance the beef market in South Korea.

## INTRODUCTION

From 1995 to 2015, the annual per capita consumption of meat and beef in South Korea increased dramatically from 27.5 kg to 51.3 kg and 6.7 kg to 11.6 kg, respectively [1]. Korean consumers prefer beef from native Korean Hanwoo cattle (*Bos Taurus coreanae*) over imported beef because they believe that Hanwoo beef has better flavor and marbling than imported beef, and they are accustomed to the taste of Hanwoo beef with its high levels of oleic acid and intramuscular fat [2,3]. In Korea, cattle carcasses are typically divided into 10 wholesale cuts: loin, tenderloin, rib, brisket, topside, shank, striploin, neck, blade, and rump [4]. Each cut has its own physicochemical properties such as taste, texture, and nutritional composition [5]. Fat content affects sensory traits such as texture, tenderness, juiciness, and flavor of beef, and consumer preferences depend on the sensory traits [6,7]. Consumer preference for various beef cuts is a major factor that affects market demand. Korean consumers generally prefer higher-fat cuts of beef over lower-fat cuts. This preference has caused the prices of high-preference cuts (HPCs) to increase well over double those of low-preference cuts (LPCs) [8]. Despite high prices for HPCs, the continuing demand for HPCs has led a market imbalance with an oversupply of LPC beef [9]. Thus, efforts to promote LPC consumption will help to increase the economic stability of the Korean beef market [9].

Along with flavor and appealing presentation, meat also must possess attributes of healthfulness, freshness, safety, functionality, and nutritional value in order to satisfy consumer preferences [10]. Health professionals worldwide have recommended decreasing overall consumption of saturated fatty acids (SFAs), trans-fatty acids, and cholesterol that cause obesity, cardiovascular diseases, and atherosclerosis, and increasing intake of ω-3 polyunsaturated fatty acids (PUFAs) [11]. Thus, promoting meat with such characteristics would help effort to improve public health.

On the other hand, meats with more unsaturated fatty acids —especially those unsaturated fatty acids with more than two double bonds—are more rapidly oxidized than meats more SFAs [12]. Lipid oxidation in meat, which results in the production of free radicals, is related to meat quality attributes such as color, texture, flavor, and nutritional value [13,14]. Meat oxidation thus can lead to loss of nutritional value and reduced sensory quality [15]. There are several mechanisms in muscle tissue for counteracting oxidation, including endogenous enzymatic antioxidant systems such as glutathione peroxidase (GPx), glutathione-S-transferase (GST), and superoxide dismutase (SOD) [16]. So far, antioxidant activities have been studied in many materials. However, no studies have been performed where different cuts of beef with the same meat quality grade were compared for fatty acid compositions and antioxidant activities. To evaluate this, comparisons of HPCs and LPCs of beef were conducted using six beef cuts (loin, tenderloin, rib, brisket, topside, and shank) from Hanwoo (*Bos taurus coreanae*) cows.

## MATERIALS AND METHODS

### Meat sample preparation

Comparisons of HPCs and LPCs in this study used the same Hanwoo carcasses and samples used for the previously published study on beef chemical composition, free amino acid contents and antioxidant activity of beef [9]. Fresh samples from three HPCs (loin, tenderloin, rib) and three LPCs (brisket, topside, shank) of 10 Hanwoo (*Bos taurus coreanae*) cows (quality grade 1, medium marbled; carcass weight 365.7±20.8 kg, min 323 kg and max 395 kg; slaughter age 57.2±10.7 months, min 34 months and max 67 months) were purchased from a wholesale market. The 10 cows, grown on different farms with different diets, were slaughtered on the same day at the same local municipal slaughterhouse (Eumseong, Korea). The carcasses were evaluated one day after slaughter using the Hanwoo beef carcass grading system of the Korea Institute for Animal Products Quality Evaluation [4], and then divided into cuts and sent to the Sungjin Co., Ltd. wholesale market (Seoul, Korea). Within any one category (e.g., all loin cuts or all shank cuts), each 500 g sample was selected as a region of muscle that included intramuscular fat from the same center part of every cut. The six muscles were *m. longissimus dorsi* (loin), *m. psoas major* (tenderloin), *m. serratus ventralis* (rib), *m. rectus abdominis* (brisket), *m. semimembranosus* (topside), and *m. superficialis flexor* (shank). The samples were transported by a cold-storage car from market to lab. Immediately after delivery, the samples from the sixty cuts of beef were dried using a lyophilizer (PVTFD 200R, Ilshin Lab Co., Ltd., Seoul, Korea) at −45°C (10 mmTorr for 72 h) and then ground into powder form using a sample grinder (HMF-3100S, Hanil Co., Wonju, Korea).

### Fatty acid analysis

To perform this experiment, total lipids from the meat samples were extracted using a chloroform:methanol mixture (2:1, v:v) including 0.01% butylated hydroxytoluene. The extracted lipids were dried using a rotary evaporator (N-110, Tokyo Rikakikai Co., Ltd, Tokyo, Japan) in vacuum and converted to methyl esters by base-catalyzed trans-esterification with sodium methoxide for 2 h at 30°C [17]. Fatty acid methyl esters (FAMEs) from meat samples were quantified using gas chromatography (GC-2010 Plus, Shimadzu, Tokyo, Japan) fused with silica capillary column (SPTM-2560, 100 m×0.25 mm i.d, 0.20-μm film thickness, Supelco, Bellefonte, PA, USA). Analysis was done using an initial isothermic period of 100°C for 4 min, followed by a temperature increase at a rate of 3°C/min to 240°C and then an isothermic period of 240°C for 10 min. One microliter of FAMEs n-hexane was injected into the column. The injection port and detector were maintained at 225°C and 285°C, respectively, with helium functioned gas. The components were identified by comparing the retention times of FAME peaks from samples with the standard (47885-U, Supelco 37 Component FAME Mix, Supelco, USA). The compositions of individual fatty acids were first quantified as mg per kg of meat using the internal standard. Then, total fatty acid contents were expressed as g per 100 g of meat, while individual fatty acid compositions were expressed as a weight percentage of the total fatty acids.

### Free radical scavenging activity measurement

Meat samples were homogenized in nine volumes of phosphate-buffered saline (PBS, pH 7.4; Gibco, Paisley, Scotland, UK). The homogenates were centrifuged (1,660 g at 4°C) for 30 min; the supernatant was used for free radical scavenging activity assays with electron spin resonance (ESR) spectroscopy, which is a direct method of measuring production of free radicals.

#### Hydroxyl radical scavenging activity

Hydroxyl radical scavenging activity was assayed by the method used by Rosen and Rauckman [18]. Hydroxyl radicals were generated via the iron-catalyzed Haber–Weiss reaction (Fenton-driven Haber–Weiss reaction), and the generated hydroxyl radicals rapidly reacted with nitrone spin-trap 5,5-dimethyl-1-pyrroline-N-oxide (DMPO). The resultant DMPO-OH adduct was detectable with an ESR spectrometer. Thirty microliters of each sample was mixed with 30 μL of DMPO (0.3 M), 30 μL of FeSO_4_ (10 mM), and 30 μL of H_2_O_2_ (10 mM) in a phosphate buffer solution (pH 7.2), and then transferred into a 100-μL Teflon capillary tube. After 2 min 30 s, the ESR spectrum was recorded using an ESR spectrometer (JEOL Ltd., Tokyo, Japan). Experimental conditions were as follows: central field, 3,475 G; modulation frequency, 100 kHz; modulation amplitude, 2 G; microwave power, 1 mW; gain, 6.3×10^5^, and temperature, 298 K.

#### Alkyl radical scavenging activity

Alkyl radical scavenging activity was measured by the method used by Hiramoto et al [19]. Alkyl radicals were generated by 2, 2′-azobis (2-amidinopropane) dihydrochloride (AAPH). Thirty microliters of each sample was mixed with 30 μL of PBS (pH 7.4), 30 μL of 40 mM AAPH, and 30 μL of 40 mM (4-pyridyl-1-oxide)-N-tert-butylnitrone. After incubation at 37°C in a water bath for 30 min, the samples were transferred to a 100-μL Teflon capillary tube. The spin adduct was recorded on an ESR spectrometer. Measurement conditions were as follows: central field, 3,475 G; modulation frequency, 100 kHz; modulation amplitude, 2 G; microwave power, 1 mW; gain, 6.3×10^5^, and temperature, 298 K.

#### 2, 2′-diphenyl-1-picrylhydrazyl radical scavenging activity

DPPH radical scavenging activity was measured using the method described by Nanjo et al [20]. Sixty microliters of each sample was added to 60 μL of DPPH (60 mM) in methanol solution. After mixing vigorously for 10 s, the solution was transferred into a 100-μL Teflon capillary tube, and the scavenging activity of each sample for the DPPH radical was measured using an ESR spectrometer. The spin adduct was measured on an ESR spectrometer exactly 2 min later. Experimental conditions were as follows: central field, 3,475 G; modulation frequency, 100 kHz; modulation amplitude, 2 G; microwave power, 5 mW; gain, 6.3×10^5^, and temperature, 298 K.

### Antioxidative enzyme activity determination

Meat samples were homogenized with ice-cold homogenizing buffer containing 50% potassium chloride, 1 M Tris-hydrogen chloride, and 0.5 M ethylenediaminetetraacetic acid (EDTA, pH 7.0) at 1:10 w/v concentration. The homogenate was centrifuged (1,660 g at 4°C) for 30 min, and the supernatant was used for enzyme assays. Protein concentration was measured using the Biuret method described by Gornall et al [21].

#### Glutathione peroxidase activity

GPx activity was determined following the oxidation of nicotinamide adenine dinucleotide phosphate (NADPH) with t-butyl hydroperoxide as a substrate [22]. The reaction mixture contained 2 mM glutathione (GSH), 2 U GSH reductase, 0.12 mM NADPH, 0.5 mM sodium azide, 0.2 mM H_2_O_2_, 3 mM cumene hydroperoxide, and 100 mM phosphate buffer (pH 7.5). The GPx activity was evaluated by monitoring the decrease in NADPH concentration at 340 nm, and expressed as nmal GPx/min. mg protein.

#### Glutathione-S-transferase activity

The activity of GST toward 1-chloro-2,4-dinitrobenzene (CDNB) was determined using the method developed by Habig et al [23]. The approach was based on the reaction of CDNB with the –SH group of GSH, which was catalyzed by the GST contained in the samples. Here, 2 μL of sample was reacted with 1 mL of the reaction mixture containing 1 mM CDNB, 1 mM GSH, and 100 mM phosphate buffer (pH 7.5). Following conjugation of the thiol group of the GSH to the CDNB substrate, there was an increase in the absorbance at 340 nm. The GST activity was expressed as μmol GSH/min. mg protein.

#### Superoxide dismutase activity

SOD activity was estimated by the method described by Misra and Fridovich [24]. The samples were mixed with 1 M xanthine, 0.2 mM cytochrome, and 0.05 M potassium cyanide in 0.05 M potassium phosphate/0.1 mM EDTA buffer; and then xanthine oxidase was added to the reaction mixture. The SOD activity was measured from the inhibition of the reduction rate of cytochrome by superoxide radical as observed spectrophotometrically at 550 nm. The SOD activity was expressed as inhibition rate percentage.

### Statistical analysis

All analyses used ten samples for each of the six cut of beef. In addition, each assay was performed three times from the sample to ensure reproducibility. The results were expressed as the mean and standard error of the mean, and statistical analysis was performed by Duncan’s multiple range test using SAS 9.3 (SAS Institute, Cary, NC, USA). The statistical significance was defined at p<0.05.

## RESULTS AND DISCUSSION

### Fatty acid compositions

The fatty acid compositions of the HPCs and LPCs from Hanwoo cows are presented in Table 1. The total fatty acid contents of loin and tenderloin were nearly double those of topside and shank, and the total fatty acid content of HPCs was significantly higher than that of LPCs (p<0.05) (Table 1). In addition, the compositions for most of the fatty acids were found to be significantly different among the six beef cuts (p<0.05) (Table 1). The percentages of total SFAs and total monounsaturated fatty acids (MUFAs) were significantly different among six beef cuts (p<0.05) (Table 1). The percentages of total PUFAs were significantly higher in LPCs than in HPCs (p<0.05) (Table 1). SFAs have hypercholesterolemic properties that are considered to be harmful to human health, whereas PUFAs can help prevent some human diseases, such as cancer, obesity, and cardiovascular diseases [25]. Since a higher ratio of PUFAs to SFAs is desirable for more healthful meat [12]. The meat composition ratio of SFAs to PUFAs could be used as an indicator for evaluating meat in terms of healthfulness.

### Free radical scavenging activities

The hydroxyl, alkyl, and DPPH radical scavenging activities of the HPCs and LPCs from Hanwoo cows shown in Table 2. All radical scavenging activities of the meat samples occurred in a dose-dependent manner. Free radical scavenging activity was expressed as the IC_50_ value, which means μg per mL concentration of sample required for scavenging 50% of free radicals. During the experiment, the meat samples showed higher scavenging activities for the hydroxyl radicals than for other radicals indicating lower IC_50_ values. The hydroxyl radical scavenging activities in LPCs were significantly higher than those in HPCs (p<0.05) (Table 2). The hydroxyl radical is one of the most reactive radicals produced from biological molecules, and it can damage living cells [26]. Moreover, LPC samples exhibited stronger alkyl radical scavenging activities than HPC samples (p<0.05) (Table 2). The DPPH radical scavenging activities of LPCs were also significantly higher than those of HPCs (p<0.05) (Table 2). DPPH, a stable free radical, has been generally used to measure the free radical scavenging capacity of various antioxidant substances [27]. Free radicals could be more prone to attack PUFAs [13]. The ratio of PUFAs to SFAs was higher in LPCs, but the amount of PUFAs was higher in HPCs (data not shown). This suggests that HPCs containing higher PUFA contents could be exposed to more free radicals, although a variety of other complex factors related to the production of and scavenging of free radicals should also be considered.

Cheong et al [28] reported IC_50_ values of hydroxyl, alkyl, and DPPH radical scavenging activity in Hanwoo steer loin (longissimus muscle) as 125, 334, and 172 μg/mL, respectively; these values are higher than the findings of the present study. Although the free radical scavenging activities of the present study were lower than those reported by other researchers, the results show that free radical scavenging activities in LPCs were significantly higher than those in HPCs, providing positive information to support LPC consumption (p<0.05). More controlled studies will be needed to investigate antioxidant activities for meat samples of various cuts and sources since antioxidant activity may depend on an animal’s sex, age, and feed.

### Antioxidative enzyme activities

The antioxidative enzyme activities of the HPCs and LPCs from Hanwoo cows are presented in Table 3. All antioxidative enzyme (GPx, GST, and SOD) activities were significantly higher in LPC samples than in HPC samples (p<0.05) (Table 3). Oxidative reactions in meat leads to a loss of quality and nutrition factors [14]. Several antioxidant systems including endogenous enzymes such as GPx, GST, and SOD have been implicated in minimizing oxidation reactions in meat [16]. The endogenous enzymes are the primary antioxidant mechanism *in vivo* [29]. GPx reduces hydrogen peroxide and lipoperoxides formed form lipid oxidation [19]. SOD defends cells against reactive oxygen species and free radicals, and scavenges superoxide anion forming hydrogen peroxide [13,16]. The results of this study support the implication of these antioxidative enzymes in the protection against oxidative damages in beef.

In conclusion, these results support the idea that LPCs of beef such as brisket, topside, and shank have better ratios of PUFAs to SFAs and higher levels of antioxidant activities than HPCs such as loin, tenderloin, and rib; such information could be useful for health-conscious consumers when selecting from among available choices of beef. In addition to being useful nutritional information, the results reported here may provide an impetus for increasing overall consumption of low-preference beef cuts, which could help to balance the beef market in South Korea.

## Figures and Tables

**Table 1:** Fatty acid compositions of high-preference and low-preference beef cuts from Hanwoo cows (n = 10)

Fatty acid (% total fatty acid)	HPCs			LPCs			SEM	p-value
	
	Loin	Tenderloin	Rib	Brisket	Topside	Shank		
Capric acid (C10:0)	0.01	0.01	0.00	0.00	0.00	0.00	0.01	0.411
Lauric acid (C12:0)	0.07	0.07	0.04	0.02	0.01	0.04	0.03	0.240
Myristic acid (C14:0)	3.33^ab^	3.63^a^	2.75^b^	2.72^b^	2.82^b^	3.00^ab^	0.25	0.005
Pentadecanoic acid (C15:0)	0.24	0.28	0.15	0.19	0.18	0.24	0.04	0.064
Palmitic acid (C16:0)	27.00^a^	27.11^a^	22.02^c^	23.93^bc^	25.97^ab^	25.20^ab^	0.96	<0.001
Heptadecanoic acid (C17:0)	0.72	0.78	0.51	0.58	0.62	0.74	0.11	0.153
Stearic acid (C18:0)	11.30	10.29	7.95	8.76	9.04	10.92	1.27	0.090
Arachidic acid (C20:0)	0.04	0.04	0.01	0.01	0.00	0.05	0.03	0.547
SFAs (total)	42.71^a^	42.21^ab^	33.45^c^	36.21^bc^	38.63^b^	40.19^ab^	1.91	<0.001
Myristoleic acid (C14:1)	1.10	1.10	1.65	1.34	1.25	0.96	0.22	0.074
Palmitoleic acid (C16:1)	5.04^b^	5.16^b^	7.39^a^	6.47^ab^	6.19^ab^	5.14^b^	0.82	0.046
Heptadecenoic acid (C17:1)	0.66	0.64	0.83	0.83	0.74	0.78	0.12	0.479
Elaidic acid (C18:1(t))	0.44	0.78	0.70	0.54	0.69	0.82	0.38	0.909
Oleic acid (C18:1(c))	47.75^b^	47.71^b^	53.26^a^	51.45^ab^	49.64^ab^	48.56^b^	1.26	<0.001
Cis-11-Eicosenoic acid (C20:1)	0.26	0.27	0.40	0.40	0.23	0.23	0.09	0.217
MUFAs (total)	55.25^b^	55.66^b^	64.23^a^	61.03^ab^	58.74^ab^	56.50^b^	1.89	<0.001
Linolelaidic acid (C18:2(t)) (ω-6)	0.15	0.16	0.17	0.14	0.10	0.14	0.04	0.550
Linoleic acid (C18:2(c)) (ω-6)	1.31^c^	1.42^c^	1.43^c^	1.75^b^	1.78^b^	2.29^a^	0.13	<0.001
Linolenic acid (C18:3) (ω-3)	0.05^c^	0.05^c^	0.06^c^	0.07^b^	0.07^b^	0.09^a^	0.01	<0.001
Eicosatrienoic acid (C20:3) (ω-6)	0.11^ab^	0.09^b^	0.15^ab^	0.21^ab^	0.17^ab^	0.23^a^	0.05	0.041
PUFAs (total)	1.63^c^	1.72^c^	1.80^c^	2.16^b^	2.12^b^	2.74^a^	0.17	<0.001
ω-6	1.58^c^	1.67^c^	1.74^bc^	2.09^b^	2.05^b^	2.65^a^	0.17	<0.001
ω-3	0.05^c^	0.05^c^	0.06^bc^	0.07^b^	0.07^b^	0.09^a^	0.01	<0.001
MUFA/SFA	1.31^c^	1.36^c^	1.92^a^	1.69^b^	1.53^bc^	1.41^c^	0.12	<0.001
PUFA/SFA	0.04^c^	0.04^c^	0.05^b^	0.06^a^	0.05^b^	0.07^a^	0.01	<0.001
ω-6/ω-3	31.95	34.75	31.67	29.90	29.22	30.96	3.78	0.736
Total fatty acid (g per 100g of meat)	5.04	4.33	3.79	3.23	2.05	2.51	1.20	0.164

SEM, standard error of the means; HPCs, high-preference cuts; LPCs, low-preference cuts; SFAs, saturated fatty acids (sum of C10:0, C12:0, C14:0, C15:0, C16:0, C17:0, C18:0, and C20:0); MUFAs, monounsaturated fatty acids (sum of C14:1, C16:1, C17:1, C18:1(t), C18:1(c), and C20:1); PUFAs, polyunsaturated fatty acids (sum of C18:2(t), C18:2(c), C18:3, C20:2, and C20:3); MUFA/SFA, ratio of monounsaturated fatty acids and saturated fatty acids; PUFA/SFA, ratio of polyunsaturated fatty acids and saturated fatty acids; ω-6, sum of C18:2(t), C18:2(c), C20:2, and C20:3; ω-3, C18:3; ω-6/ω-3, ratio of ω-6 and ω-3 fatty acids.

a–d Means in the same row with no superscript letters after them or with a common superscript letter are not significantly different (p<0.05).

**Table 2:** Hydroxyl, Alkyl, and DPPH radical scavenging activities of high-preference and low-preference beef cuts from Hanwoo cows (n = 10)

Radical scavenging activity (IC_50_ μg/mL)	HPCs			LPCs			SEM	p-value
	
	Loin	Tenderloin	Rib	Brisket	Topside	Shank		
Hydroxyl	679^a^	715^a^	606^b^	456^c^	452^c^	493^c^	4.09	<0.001
Alkyl	886^a^	753^c^	808^b^	621^d^	642^d^	526^e^	4.39	<0.001
DPPH	2,432^b^	2,589^a^	2,315^b^	1,962^c^	1,742^d^	1,862^cd^	12.35	<0.001

DPPH, 2,2′-diphenyl-1-picrylhydrazyl; HPCs, high-preference cuts; LPCs, low-preference cuts; SEM, standard error of the mean.

a–e Means in the same row with no superscript letters after them or with a common superscript letter are not significantly different (p<0.05).

**Table 3:** GPx, GST, and SOD activities of high-preference and low-preference beef cuts from Hanwoo cows (n = 10)

Enzyme activity	HPCs			LPCs			SEM	p-value
	
	Loin	Tenderloin	Rib	Brisket	Topside	Shank		
GPx (mmoles/min. mg protein)	1.05^b^	1.02^b^	1.03^b^	1.37^a^	1.39^a^	1.40^a^	0.01	<0.001
GST (μmoles/min. mg protein)	20.46^b^	17.00^c^	19.88^b^	24.96^a^	24.87^a^	25.22^a^	0.25	<0.001
SOD (% inhibition)	88.36^b^	85.75^b^	85.58^b^	94.70^a^	94.70^a^	94.66^a^	0.67	<0.001

GPx, glutathione peroxidase; GST, glutathione-*S*-transferase; SOD, superoxide dismutase; HPCs, high-preference cuts; LPCs, low-preference cuts; SEM, standard error of the means.

a–c Different superscript letters indicate significant differences among beef cuts within any row (p<0.05).
